# A Fraud Exposed

**Published:** 1887-11

**Authors:** 


### A FRAUD EXPOSED.

In the *New York Medical Record* for July 30th, 1887, appeared an article by Dr. J. Herbert Claiborne, Jr., announcing the discovery by Drs. Seward and Goodman of a new local anæsthetic. The story told of its discovery is that Dr. Goodman, while traveling in Louisiana, less than a year ago, wished to make a poultice to apply to a swelling on his horse's leg; not having flax seed meal, or other materials used in making poultices at hand, he gathered up some leaves, steeped them in very hot water, and applied them to the swelling. Later he incised the swelling freely, and was surprised to find that the horse gave no evidence of pain during the operation. Thinking this might possibly be due to some anæsthetic property residing in the leaves, he made another similar poultice a few weeks later, and applied it to an inflamed bursa on another horse. After waiting a short while he opened the bursa with his knife, and the horse did not wince at all. Here, then, was a great discovery. It possessed in an eminent degree the elements of simplicity and accident, which characterize nearly all great discoveries.

Dr. Goodman sent some of the leaves to Dr. Seward, of New Jersey, who succeeded in isolating an alkaloid possessing anæsthetic properties.

Dr. J. Herbert Claiborne, Jr., of New York, obtained from Dr. Seward a small quantity of a 2 per cent. solution of the alkaloid, and made various experiments with it on the eye and other parts of the body.

All of his tests of the remarkable drug led irresistibly to one conclusion, viz, that stenocarpine or gleditschine (as the new alkaloid was called) possessed in a wonderful degree the power of inducing local anæsthesia. In addition to its anæsthetic property, it would also produce marked dilatation of the pupil when instilled into the eye. In other words, it combined the properties of both cocaine and atropia. Dr. Claiborne's article naturally created a great sensation in the medical world, and was speedily followed by a contribution from the pen of Dr. H. Knapp, of New York, in the *Medical Record*, August 13th. Dr. Knapp, having succeeded in obtaining some of Dr. Claiborne's solution, made physiological, toxicological and therapeutical experiments, reaching practically the same conclusions published by Dr. Claiborne·

Nothing more appeared on the subject until October 1st, when Dr. Claiborne published an elaborate article in the *Medical Record*. From it we learn that stenocarpine or gleditschine (as Dr. C. preferred to call it) was obtained from the tree called *gleditschia triacanthos* by botanists, but commonly known throughout the South as the thorn or honey locust. Dr Claiborne still maintained the conclusions announced in his first article.

Up to this time all of the alkaloid in the world was that made by Dr. Seward, and all of the experiments of Dr. Claiborne and Dr. Knapp had been performed with a solution obtained from Dr. Seward.

An analysis of some of this same solution was made in the laboratory of Messrs. Parke, Davis & Co., and it was found to contain six per cent. of cocaine and another drug supposed to be sulphate of atropia.

F. A. Thompson, Ph. D., with much care obtained a small amount of an amorphous alkaloid from the leaves of *gleditschia*

*triacanthos*, but it was found to be totally devoid of the properties ascribed to it.

Messrs. Parke, Davis & Co. deserve the thanks of the profession for exposing this fraud. It is at present impossible to say who was the perpetrator of this hoax. Certainly the high standing of Drs. Claiborne and Knapp will prevent any suspicion attaching to them; it is our belief that they have been more victimized than the profession at large, for they are both most honorable gentlemen, and it must be exceedingly mortifying to them to have their names connected in any way with such an imposture.

What they thought to be a solution of stenocarpine was in realty simply a mixture of cocaine and atropia. It is in order for Drs. Goodman and Seward to rise and explain.

